# The Role of Parental Qualities in Supporting Children with ADHD

**DOI:** 10.3390/children12070845

**Published:** 2025-06-27

**Authors:** Galia Ankori, Maly Solan, Sarit Plishty, Anat Brunstein Klomek, Alan Apter, Yaron Yagil

**Affiliations:** 1 Tel-Hai College, Upper Galilee 1220800, Israel; 2 Child and Adolescence Mental Health Clinic of Maccabi Health Services, Netanya 4231111, Israel; 3 School of Medicine, Faculty of Medical & Health Sciences, Tel Aviv University, Tel Aviv 6997801, Israel; 4 School of Psychology, Reichman University, Herzliya 4610101, Israel; 5 Department of Child and Adolescent Psychiatry, Schneider Medical Center, Petah Tikva 49202, Israel

**Keywords:** mental health, parenting, mindfulness, attachment, child behavior checklist, acceptance–rejection, ADHD

## Abstract

Objective: This cross-sectional study examined assumptions about the role of parenting qualities in predicting child problems. Background: Children with ADHD often experience distress, partially linked to less adaptive parenting practices. Our working assumptions are that: parental mindfulness, insecure parent attachment styles, and parental child rejection have a significant impact upon the severity of child problems and therefore should be addressed in parental training. Methods: A total of 122 Israeli parents (55 fathers (Mage = 43.8; SD = 4.01) and 67 mothers (Mage = 41.6; SD = 4.59)) of 75 children with attention deficit hyperactivity disorder (ADHD) (Mage = 8.4; SD = 1.56) completed self-report measures: the Experience of Close Relationships scale (ECR), the Mindfulness Attention Awareness Scale (MAAS), the Parental Acceptance–Rejection Questionnaire (PARQ), and Achenbach Child Behavior Checklist (CBCL). Data were analyzed using descriptive statistics, Pearson correlations, and structural equation modeling (SEM). Results: The key finding was that a latent ‘parental rejection/non-warmth’ factor mediated the relationship between (a) parents’ anxious attachment and child behavior problems, and (b) parental mindfulness and child problems. Parental rejection emerged as the strongest predictor of child difficulties. Conclusions: Parental training for parents of children with ADHD should prioritize reducing rejection while also addressing mindfulness and anxious attachment style to promote child well-being. Clinical Trial Registration: Group training for parents whose children suffer from ADHD and comorbidity using a behavioral-dynamic approach (SPBT). Registered at Veeva Vault.

## 1. Introduction

Mental health interventions for children can be conducted directly with the child, with the family [1], or in the format of parent-training programs [2]. The first option assumes that therapy should concentrate on alleviating and regulating the child’s distress through direct intervention [3]. The latter two rely on the assumption that children’s distress originates from family-as-system malfunctions [4], or that maladaptive parenting intensifies children’s behavioral problems [5]. There is an extensive body of research that corroborates the effectiveness of parenting programs [2,6]. Parent-training groups have been found effective for children exhibiting both externalizing behaviors and internalizing problems [5,7]. In line with these findings, we adopted the practice of parent-training groups as one of the central intervention modalities at our health maintenance organization (HMO) community child and adolescent mental health clinic. In one of these groups, that of parents to children diagnosed with attention deficit hyperactivity disorder (ADHD), we focus on improving parent–child relationships through increasing parental mindfulness, expanding parents’ insight into their attachment-style-derived behaviors, improving their parental acceptance, and minimizing the rejection of the child. By doing so, we expect to decrease child problems.

This intervention is grounded in three postulations: (a) higher levels of parental mindfulness are associated with lower levels of child problems; (b) insecure parent attachment styles (anxious or avoidant) are associated with higher levels of child problems; and (c) lower levels of parental child rejection are associated with fewer child problems. The current study aims to systematically validate these assumptions, by reviewing previous research and conducting an empirical investigation within a clinical sample drawn from our practice.

The term ‘mindfulness’ entails the ability to (a) consciously and purposely monitor inner and outer environments, (b) focus awareness on a selected range of experiences, and (c) reorient awareness and attention to the current experience, in a wholeheartedly receptive manner [8]. Mindfulness is also described as “a moment-to-moment awareness of one’s experience without judgment”, and practicing it may yield affective, interpersonal, and intrapersonal benefits [9] (p. 198).

Wheeler et al. [10] highlighted the significance of differentiating between dispositional mindfulness (trait mindfulness) and deliberate (intentional) mindfulness. Tang & Braver [11] further explained that although mindfulness is an intentional practice, pre-existing differences in dispositional mindfulness might affect its practice. Therefore, in the current study, we choose to relate to mindfulness as a trait that can be further enhanced through training [11].

Mindfulness has been suggested as relevant to parenting skills due to its contribution to attentive listening abilities, compassion, nonjudgmental acceptance, emotional awareness of self and child, and parental self-regulation [12]. It has also been argued that mindful parenting reduces parental stress and reactivity, preoccupation with psychopathology, and the intergenerational transmission of dysfunctional patterns, while improving parental executive functioning, self-care, marital functioning, and co-parenting [13]. Empirical findings demonstrate an association of high-leveled mindfulness with reduced parental stress [14], improved parental well-being [15], increased control over parental escalating behaviors, and improved emotional regulation [16]. Parental mindfulness has also been found to affect parent–child relationships by (a) increasing parents’ awareness of their children’s emotions [14], (b) decreasing parental negative feelings towards their children, and (c) lessening parental submission to children’s demands [16]. Finally, several studies have demonstrated that parents’ mindfulness was associated with decreased internalizing and externalizing disorders among their children [17,18,19], enhanced compliance with parental demands [20], decreased negativity of the child towards the parent [21], improved well-being, and more secure perceptions of the child–parent relationship among children [22]. Based on this review, we hypothesize that children of parents characterized by a higher level of mindfulness would exhibit fewer problems.

According to Bowlby, attachment behaviors draw infants and mothers into close proximity for survival. When facing physical or emotional threats, individuals activate their attachment system, turning to internalized representations or real supportive figures to ease distress [23]. Through these “being-cared-for experiences”, infants develop self-soothing and emotional regulation capacities [24]. Secure attachment supports flexible and effective emotion regulation, while insecure styles may hinder it [25].

Ainsworth identified three attachment styles, with Main & Solomon later adding a fourth: secure, anxious, avoidant, and disorganized [23,26]. Levy and Davis [27] further conceptualized these using a two-dimensional model: avoidance (discomfort with closeness) and anxiety (fear of abandonment). Secure individuals (low avoidance, low anxiety) seek support in distress. Anxious individuals (low avoidance, high anxiety) seek comfort but fear abandonment. Avoidant individuals (high avoidance, low anxiety) resist seeking comfort. Disorganized individuals (high in both dimensions) feel discomfort in relationships yet remain dependent [28].

Secure attachment is crucial for child development, influencing emotional regulation, exploration, relationships, support-seeking, cognitive and academic success, life satisfaction, and conflict resolution [29,30,31,32,33,34]. This study focuses on parental attachment styles and their impact on children. Bretherton [35] argued that unresolved rejecting or neglectful childhood attachments lead to insensitive parenting, affecting children’s self-perception and internal working models.

Research supports these connections. Borelli et al. [36] found that children of highly anxious parents showed more negative emotions, while those with avoidant parents showed fewer, indicating deactivation. Sümer and Harma [37] linked avoidant parental attachment to children’s increased trait anxiety and lower academic self-efficacy. Mothers’ anxiety predicted boys’ trait anxiety, while fathers’ avoidance predicted girls’. Esbjørn et al. [38] found that maternal anxiety and both forms of paternal insecurity predicted anxiety-related disorders in children. Such effects likely stem from parents’ unresolved attachment issues, as previously suggested [35]. Based on this, we hypothesized that parents’ insecure attachment (avoidant and anxious) would positively correlate with children’s distress.

Rohner’s [39] parental acceptance–rejection theory posits that children experience varying levels of acceptance (e.g., warmth, care, and support) or rejection, shaped by consistent parental behavior patterns. Rejection can be experienced in any combination of three parental attitudes: (a) hostile/aggressive, (b) indifferent/neglecting, and (c) undifferentiated rejection (absence of overt neglect or aggression, but the child experiences no true parental care).

Children who experience significant parental rejection often feel resentment, hostility, and other destructive emotions. Some scholars argue that when emotional needs for closeness are consistently unmet, rejection constitutes emotional abuse [40,41]. Rejected children may become hypervigilant and emotionally unresponsive to guard against further hurt [39]. Perceived parental rejection is linked to psychological maladjustment [42], depression [43], fear of intimacy, and diminished trust in others [44]. It also correlates with externalizing behaviors (e.g., hostility) [35,41] and internalizing symptoms such as anxiety, low self-esteem, emotion suppression, and generalized anxiety disorder [40,41].

Scanlon and Epkins [45] found that both mother-reported and child-perceived maternal rejection predicted higher child-reported depression and anxiety. Rohner et al. [39] noted that rejection increases risk for insecurity, depression, anxiety, low self-esteem, and substance use, accounting for about a quarter of variance in psychological adjustment across cultures. Vučković et al. [46] also reported associations between parental rejection and externalizing behaviors in school-aged children. Similarly, a recent cross-cultural study found that both maternal and paternal rejection predicted internalizing and externalizing problems in children aged 7–14 [47]. Based on this evidence, we hypothesized that children exposed to higher parental rejection would exhibit greater psychological distress.

Several studies suggest that parental mindfulness, as a predictor, may influence levels of distress among children through the following mediating variables: parenting quality [21], positive parenting practices [48], parental acceptance [49], perceived maternal warmth [50], and non-rejection [51].These parental attitudes and practices were found to improve children and adolescents’ emotional regulation, as well as decrease negativity [21], internalizing and externalizing behaviors [48], and other emotional problems [50]. Based on this evidence, we hypothesize that parental acceptance–rejection mediates the relationship between parental mindfulness and child outcomes: mindfulness fosters more accepting parenting, which then promotes better psychological adjustment in children.

Similarly, previous work has linked parents’ attachment styles with variations in parenting behavior [52,53,54]. Securely attached parents tend to be more emotionally available and less rejecting. Kilmann et al. [55], for instance, found that securely attached mothers were perceived as more accepting by their daughters. These findings suggest that parental acceptance–rejection may mediate the relationship between parental attachment style and children’s psychological outcomes, serving as the mechanism through which attachment influences parenting and, consequently, child adjustment.

## 2. Material and Methods

### 2.1. Participants

This study was conducted in a regional HMO child and adolescent mental health clinic that serves urban and rural populations, with an average of approximately 2500 referrals per year. The data for the current report was drawn from a longitudinal prospective study (Trial NCT02824796), designed to evaluate outcomes of parent-training groups (PTGs). The current report relied on baseline measurements that were collected during the 2016–2019 calendar years, with a clinical sample (n = 153) of children diagnosed with ADHD, who were deemed eligible to participate in the parental training groups and the study. None of them demonstrated exclusion criteria: signs of schizophrenic spectrum and other psychotic disorders, signs of major affective disorders, signs of intellectual developmental disorders, or signs of autistic spectrum disorders. Out of the eligible children, 75 children (49%) were ultimately included, after having excluded children whose parents could not commit to the entire program. Data were collected from 122 parents (55 fathers and 67 mothers) who provided informed consent and completed baseline assessment questionnaires (see Table 1).

### 2.2. Data Collection

The participants were given four questionnaires to complete: three parental characteristics self-report measures (ECR, MAAS, and PARQ), and one parent report of child problem severity (CBCL).

The Experience of Close Relationships scale (ECR) [27] is a 36-item, Likert-type self-report measure meant to evaluate parents’ attachment-style on two subscales: avoidance and anxiety. Participants are asked to state the degree to which they find the descriptions in the items to match their own thoughts, feelings, and behaviors (1 = disagree; 7 = agree). The ECR was translated to Hebrew by Mikulincer & Florian [56], and validated (Cronbach’s *α* > 0.88 for both subscales) by Findler et al. [57]. In the current study, the Cronbach’s *α* was 0.86 for the avoidance scale, and 0.88 for the anxiety scale.

The Mindfulness Attention Awareness scale (MAAS) [58] is a 15-item self-report Likert-type (1 = almost always; 6 = almost never) measure. It assesses the frequency of informed sensitive awareness to attention (e.g., “I find it difficult to stay focused on what’s happening in the present”). The MAAS was translated to Hebrew by Kinori [59] (Cronbach’s *α* = 0.87). Its reliability in the current study was Cronbach’s *α* = 0.88.

The Parental Acceptance–Rejection Questionnaire (PARQ) [60] is a 60-item, four-degree Likert-type self-report measure (1 = almost always; 4 = almost never), in which parents are asked to reflect and report their current accepting-rejecting and controlling behaviors towards their children. This measure consists of four scales: (a) warmth and affection, (b) hostility and aggression, (c) indifference and neglect, and (d) undifferentiated rejection. The PARQ was translated to Hebrew by Kinori [59] (Cronbach’s *α* = 0.79, 0.89, 0.70, and 0.71, respectively). In the current study, Cronbach’s *α* was 0.87, 0.87, 0.76, and 0.82, respectively. We also tested the hypothesis that parental acceptance–rejection moderates or mediates the relationship between parental characteristics and child problems. For this purpose, we created, using the SEM model, a latent variable which accounts for the three non-warm attitudes (latent parental non-warm factor). It should be noted that we found high Pearson correlations between the three of them (see Table 2).

The Achenbach Child Behavior Checklist (CBCL) [61,62] is a standardized 113-item form that parents, teachers, and children fill out to assess the child’s behavioral and emotional competencies and problem manifestations in the past six months. Respondents are asked to select one of three possible answers: 0 = never; 1 = sometimes; 2 = always. CBCL items are grouped into eight categories. The first three categories (anxious-depressed, withdrawn-depressed, and somatic complaints) are grouped into a higher-order scale named internalizing problems. The following two (rule breaking behavior and aggressive behavior) are grouped into the higher-order scale named externalizing problems. The last three categories are social problems (i.e., dependent, lonely, and clumsy), thought problems (i.e., harms self, hears things, and repeats acts), and attention problems (i.e., fails to finish, daydreams, and impulsive). Finally, the CBCL suggests a ninth category which includes other problems (i.e., cruel to animals, overeats, and wets bed). CBCL scores are presented in three forms, namely, as completed by the respondents, *t*-scores, and percentile scores relative to the unique distribution of each competency or problem. In the current article, we report the *t*-scores. Each problem/competency category was interpreted in relation to three ranges: normal range (50 < *t* < 65 = percentiles 50–93), borderline range (65 < *t* < 70 = percentiles 93–98), and clinical indication (*t* > 70 = percentiles > 98). The Hebrew version of the CBCL is a well-established screening instrument used widely in clinical and research settings [63]. In the current study, we used the parent-report version regarding children aged 4 to 18.

### 2.3. Data Analysis

Statistical computations were made using the IBM statistics software (version 28.0.1.1). Sociodemographic and medical background data were processed using descriptive statistics. The measures were tested for their reliability (Cronbach’s alphas). R software (2023.12.1) was used for structural equation modeling (SEM) using lavaan package. The model included two latent variables. The latent parenthood factor encompassed hostile/aggressive, indifferent/neglecting, and undifferentiated rejection behaviors, but excluded warmth and affection. The latent child behavior problem factor included internalizing, externalizing, and other behavioral problems.

### 2.4. Procedure

This study was approved by the IRB at the medical institute in which it was conducted (Ref: 2015096) and was conducted in accordance with good clinical practice guidelines. Intake-phase data were collected after providing detailed explanations about the study’s aims, procedures, and requirements, and obtaining a signed informed consent form from both parents. Participants were asked to respond to the data collection measures during their visit to the clinic, using a computer, or in their own free time, on a secured network website.

## 3. Results

To test the direct and indirect paths from parental mindfulness and attachment styles to child behavior problems via the latent parental non-warm factor, we conducted structural equation modeling (SEM) using R (lavaan package). To account for potential covariations among the mediators and among the outcome variables, we modeled these covariations. Parent age and gender, as well as child age and gender, were included as covariates. The model provided a good fit to the data (*x*^2^(40) = 44.18; *p* = 0.299; *x*^2^/*df* = 1.10; TLI = 0.986; CFI = 0.990; RMSEA [90% C.I.] = 0.030 [0.000, 0.073]; SRMR = 0.047). When we added the warmth/affection attitude to the model, it had the same associations between the variables as in the preferred model but had a bad model fit. Moreover, the preferred model was significantly better than the model with warmth in it.

**Figure 1:**
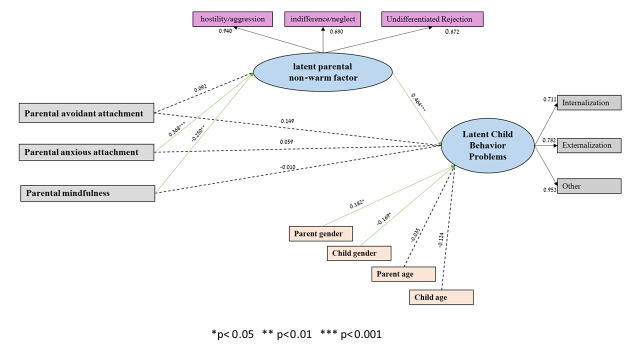
Direct and indirect effects in SEM linking parental attachment styles and mindfulness to child behavior problems.

The results indicate that the latent parental non-warm factor was predicted negatively by parental mindfulness and positively by parental anxious attachment. The association between parental avoidant attachment and the latent parental non-warm factor was insignificant. In addition, the child latent behavior problems factor was predicted positively by the latent parental non-warm factor and parent gender factor and negatively by the child female factor. Mothers (versus fathers) reported higher levels of child problems and girls (versus boys) were reported with lower problem levels. The direct paths from parents’ attachment styles and mindfulness to child behavior problems were insignificant (see Table 3 and Figure 1).

In order to test the significance of the indirect effects in the analysis bootstrapping technique, utilizing 5000 resamples, was employed to generate 95% confidence intervals. Indirect effects in which zero is not included in the 95% CI indicate a significant effect at α < 0.05. Tests of the indirect effects of the anxious attachment style on the child latent behavior problem factor, via the latent parental non-warm factor, were significant (B = 1.315; S.E = 0.436; CI; 0.460–2.171). Tests of the indirect effects of the avoidant attachment style on child latent behavior problems, via the latent parental non-warm factor, were insignificant (B = 0.328; S.E = 0.305; CI; −0.269–926). Tests of the indirect effects of parents’ mindfulness on child behavior problems via the latent parenthood factor were significant (B = −0.996; S.E = 0.414; CI; −1.808–0.184). Thus, the latent parental non-warm factor mediated the association between (i) parents’ anxious attachment style and child behavior problems and between (ii) parental mindfulness and child behavior problems. That is, a higher anxious attachment style predicts higher non-warm attitudes and behaviors in parents, which, in turn, predicts higher child behavior problems. On the other hand, higher mindfulness predicts lower non-warm attitudes and behaviors in parents, which, in turn, predicts lower child behavior problems.

## 4. Discussion

The primary aim of the current study was to empirically examine three clinical assumptions that underpin the parent training groups we offer for families of children diagnosed with ADHD. The first was that by improving parental mindfulness, one may alleviate child problems. The second was that by working upon insecure parent attachment styles, child problems can be alleviated, and the third was that assisting parents in achieving higher levels of parental child acceptance will lead to lower levels of child problems. In addition, we tested the nature of the associations between these qualities (direct vs. indirect).

Within our clinical sample, the analysis revealed that parental acceptance–rejection was the factor most strongly and directly associated with child difficulties, whereas mindfulness and parental attachment style demonstrated only indirect associations. In relation to previous knowledge, our results further underscored the role of parental acceptance–rejection in children’s well-being. It replicated the findings that were reported by Rohner et al. [64] and by Rothenberg et al. [47], in which a significant portion of the variance in children’s psychological adjustment as well as internalizing and externalizing problems was accounted for by parental acceptance–rejection. Rothenberg et al. further claimed that parental acceptance–rejection plays a major role regardless of culture, ethnicity, or geographic location. From this perspective, our study expands this claim to encompass an additional population.

Although previous research has demonstrated a direct association between parental mindfulness and a reduction in children’s internalizing and externalizing symptoms [18,19], as well as improvements in parental well-being [22], in the current study, this relationship is indirect. Rather than exerting a direct influence on child outcomes, mindfulness appears to affect them through increased parental acceptance, which serves as a mediating mechanism. This finding is similar to previous studies in which mindfulness was connected to child symptoms through parental acceptance [40] as well as similar qualities, such as better parenting quality [21], positive parenting practices [48], perceived maternal warmth [50], and non-rejection [51]. This insight contributed to a more nuanced understanding of how mindfulness functions in parenting contexts, underscoring the role of parental acceptance as a key pathway through which mindfulness achieves its benefits.

Similarly, while prior studies have identified a direct link between parental anxious attachment and elevated child difficulties [36,37,38], our findings indicate that this effect is also mediated by parental acceptance. That is, anxious attachment does not seem to influence child symptoms directly but rather exerts its impact by diminishing the parent’s capacity for acceptance. These findings suggest that fostering parental acceptance may buffer against the negative effects of insecure attachment. Unlike in the case of mindfulness, in the case of attachment, our literature review did not yield findings similar to ours. Nevertheless, some previous studies demonstrate the influence of attachment style on various facets of parenting, such as parenting styles, parental involvement, and risk of child abuse [44,45], as demonstrated by Kilmann et al. [46].

Finally, in contrast to earlier findings by Sümer and Harma [37] and Esbjørn et al. [38], the parental avoidant attachment style in our study was not significantly associated with child outcomes—neither directly nor indirectly. This finding aligns with Borelli et al. [36], who found that children of parents higher in avoidant attachment reported fewer negative emotions and displayed reduced emotion-regulation efforts. Borelli et al. interpreted these results in light of a deactivating effect: parents with avoidant attachment styles tend to minimize or discourage the expression of negative affect in their children. Consequently, children learn to dampen awareness and expression of distress. Thus, rather than reflecting genuine emotional well-being, this finding may reflect this learned emotional suppression which leads to fewer detectable symptoms.

### 4.1. Clinical Implications

These findings suggest several clinical implications. We conclude that parental acceptance is the main parental quality to be targeted in our parenting training program. Notably, our literature review revealed a scarcity of parenting programs that explicitly prioritize the enhancement of parental acceptance. Nevertheless, we identified several interventions that emphasize the reduction in coercive disciplinary practices [65] or parental criticism [66], often framed within the promotion of positive parenting practices. Additionally, we contend that the potential benefits of programs incorporating mindfulness should not be underestimated, with several interventions demonstrating promising outcomes [12,13,14,16,19,67]. Similarly, attachment-based approaches that aim to revise parents’ internal working models—particularly those shaped by disorganized attachment experiences—are increasingly integrated into therapeutic settings [35]. In our own program, we address parental attachment patterns using schema therapy and offer mindfulness training components to support reflective functioning and emotional regulation [68].

### 4.2. Study Strengths and Limitations

The current research was designed to answer the question whether there is a theoretical and empirical justification for focusing on three specific parental qualities—mindfulness, insecure attachment style, and acceptance–rejection—in parent training groups. While prior research has addressed these variables individually, their combined influence, particularly in clinical interventions for families with children diagnosed with ADHD, remains underexplored. This study contributes original insight by integrating these dimensions and examining their relative impact. Notably, it identifies parental acceptance–rejection as the most salient predictor of child outcomes, offering a clear target for intervention. In doing so, it empirically clarifies key pathways within the parent–child dynamic, thereby enriching the foundational knowledge base in family science. This research further stands out by providing a critical, practice-based evaluation grounded in a real-life clinical setting—bridging theoretical postulations with applied therapeutic processes. Additionally, it sheds light on the nuanced, direct, and indirect ways parental characteristics shape child well-being, highlighting implications for tailored intervention strategies. Unlike some of the previous studies, which were conducted in controlled environments, this study reflects the complexities and constraints of actual therapeutic settings, enhancing its ecological validity and practical relevance.

The present study has two main limitations that warrant consideration. First, regarding external validity, the study was conducted within a single regional HMO and included a relatively homogeneous sample—primarily middle-class, working parents. While these participants were seeking support for substantial challenges related to their child’s ADHD, which may reflect broader patterns among similarly distressed parents, caution should be taken when generalizing the findings to more diverse socioeconomic or cultural groups. Future research is needed to examine whether these dynamics hold across varied clinical and community settings.

Second, the findings are based solely on parental self-report measures. This reliance introduces potential bias, particularly in assessing child behavior problems via the CBCL. For instance, structural equation modeling revealed that mothers tended to report higher levels of child difficulties compared to fathers. This discrepancy may reflect subjective perceptions rather than objective differences. Comparable biases have been documented in previous studies, such as Parent et al. [18], who found that parental ratings on the CBCL often exceeded children’s own self-assessments.

### 4.3. Future Research and Clinical Directions

An important next step would be to examine whether changes in children’s reported problems are correlated with changes in parental qualities over time. Relevant data have already been collected in a previously reported study [60]. This data requires reprocessing in order to test this specific research question—through δ (change) calculations, correlation analyses, and a more robust examination of the temporal and directional patterns suggested by the SEM.

In terms of clinical directions, a significant gap remains in parent training programs regarding the direct cultivation of parental acceptance toward the child. Future research should explore innovative strategies to promote this construct more explicitly and systematically. Positioning parental acceptance as a core therapeutic goal may enhance the effectiveness of existing interventions, especially when integrated with mindfulness-based and attachment-informed practices. We propose that this multidimensional approach—emphasizing parental acceptance alongside mindfulness and attachment principles—be implemented not only in healthcare settings but also adapted for broader application in educational contexts that support children and families.

### 4.4. Key Takeaways

#### 4.4.1. Clinical Focus—Prioritize Acceptance

Parental acceptance–rejection emerged as the most significant predictor of child behavioral problems, highlighting it as a primary target for intervention with parents of children with ADHD.

#### 4.4.2. Indirect Influence—Mindfulness and Attachment

Mindfulness and attachment styles impact child outcomes indirectly via parental acceptance, underscoring their supportive role in enhancing parent–child dynamics.

#### 4.4.3. Program Design—Address Emotional Dimensions

Parenting programs should move beyond behavior management to explicitly foster emotional warmth and reduce rejection.

#### 4.4.4. Research Needs—Track Change over Time

Longitudinal studies are needed to assess how changes in parental traits affect child outcomes and to test generalizability across diverse populations.

#### 4.4.5. Broader Application—Beyond Clinics

The findings support adapting acceptance-focused approaches for educational and community settings to support families more broadly.

## Figures and Tables

**Table 1:** Descriptive characteristics of participants (*n* = 122).

Variable	Values	Findings
Gender of reporting parent	Male	55 (45.1%)
	Female	67 (54.9%)
Age of reporting parent	Fathers	43.8 ± 4.01
	Mothers	41.6 ± 4.59
Profession of reporting parent	Liberal profession and Managers	27 (22.1)
	Non-academic workers	41 (33.6%)
	Academic employees	43 (35.2%)
	Missing data	11 (9.0%)
Child’s gender	Male	54 (72%)
	Female	21 (28%)
Child’s age	Mean ± SD	8.4 ± 1.56
Tentative diagnosis following Intake	ADHD	75 (100%)
Additional diagnoses	Anxiety, ODD, Behavior dis, learning dis.	23 (30%)
Higher order CBCL scores (parent’s report)		
Internalizing problems	Beyond normative range	20 (26.7%)
Externalizing problems	Beyond normative range	35 (46.7%)
Other problems	Beyond normative range	31 (41.3%)

**Table 2:** Pearson’s correlations between the three non-warm attitudes of the PARQ.

	Indifference/Neglect	Undifferentiated Rejection
Hostility/aggression	0.623 **	0.819 **
Indifference/neglect	**--**	0.586 **

* *p* < 0.05; ** *p* < 0.01.

**Table 3:** Direct and indirect effects in SEM linking parental attachment styles and mindfulness to child behavior problems.

Predicted Variable	Predicting Variable	B	SE	LLCI	ULCI
Direct effects					
Child behavior problems	Latent parenthood factor	7.600	1.703	4.263	10.937
	Avoidant attachment	1.140	0.618	−0.072	2.352
	Anxious attachment	0.454	0.610	−0.742	1.650
	Mindfulness	−0.088	0.749	−1.556	1.381
	Parent female	2.730	1.244	0.292	5.168
	Child female	−2.798	1.402	−5.546	−0.050
	Parent age	−0.058	0.169	−0.389	0.273
	Child age	−0.585	0.387	−1.343	0.172
Latent parenthood factor	Avoidant attachment	0.043	0.037	−0.030	0.117
	Anxious attachment	0.173	0.037	0.100	0.246
	Mindfulness	−0.131	0.044	−0.218	−0.045
Indirect effects					
Avoidant attachment > Parenthood > Child Behavior		0.328	0.305	−0.269	0.926
Anxious attachment > Parenthood > Child Behavior		1.315	0.436	0.460	2.171
Mindfulness > Parenthood > Child Behavior		−0.996	0.414	−1.808	−0.184

## Data Availability

The original contributions presented in this study are included in the article. Further inquiries can be directed to the corresponding author.
